# Inhalation anesthesia and total intravenous anesthesia (TIVA) regimens in patients with obesity: an updated systematic review and meta-analysis of randomized controlled trials

**DOI:** 10.1186/s44158-025-00234-1

**Published:** 2025-03-18

**Authors:** Sabrina Soledad Domene, Daniela Fulginiti, Antonia Thompson, Vanessa P. Salolin Vargas, Laura C. Rodriguez, Meraris D. Tolentino Colón, Mariela D. Fermin Madera, Juan N. Layton, María I. Peña Encarnación, Victor S. Arruarana, Camila Sanchez Cruz, Ernesto Calderon-Martínez

**Affiliations:** 1 Universidad Nacional de Mar del Plata, Mar del Plata, Argentina; 2 Pontifical Catholic University of Argentina, Buenos Aires, Argentina; 3 Stevens Institute of Technology, Hoboken, USA; 4 Facultad de Medicina, Universidad Westhill, Mexico City, Mexico; 5 Universidad de Oriente, Nucleo Bolivar, Bolivar, Venezuela; 6 Universidad Iberoamericana (UNIBE), Santo Domingo, Dominican Republic; 7 Instituto Tecnológico de Santo Domingo (INTEC), Santo Domingo, Dominican Republic; 8 Universidad del Rosario, Bogota, Colombia; 9 Brookdale University Hospital Medical Center, New York, USA; 10 Universidad Nacional Autonoma de Mexico, Mexico City, Mexico

**Keywords:** Anesthesia, Total intravenous, Postoperative nausea and vomiting, Obesity, Intraoperative vital signs, Inhalational anesthesia

## Abstract

**Background:**

Obesity is a global epidemic, projected to affect 4 billion people by 2035. Anesthesia regimens, including volatile anesthetics and total intravenous anesthesia (TIVA), impact postoperative outcomes, particularly in obese patients who face increased risks of complications. Volatile anesthetics are often associated with higher rates of postoperative nausea and vomiting (PONV), while TIVA may improve recovery but can increase costs and present additional challenges. This systematic review and meta-analysis evaluate the effects of these anesthesia methods on perioperative outcomes, including hemodynamic stability, recovery, and PONV, in this high-risk population.

**Methods:**

Adhering to the Preferred Reporting Items for Systematic Reviews and Meta-Analyses guidelines and registered in PROSPERO (CRD42024547776) studies were identified through PubMed, Web of Science, Scopus, China National Knowledge Infrastructure, CINDAHL, Cochrane, EMBASE, and Google Scholar. Two reviewers independently extracted data and assessed the risk of bias. A meta-analysis using a random-effects model was conducted.

**Results:**

Thirteen studies with 1072 participants were included. Inhalational anesthesia significantly increases PONV (RR, 2.09; 95% CI, 1.21–3.60; *p* = 0.01; *I*^2^ = 34%) and intraoperative heart rate (MD, 3.49; 95% CI, 0.01–6.97; *p* < 0.01; *I*^2^ = 67.6%) compared to TIVA. Other outcomes, including mean arterial pressure, duration of intensive care unit stay, recovery time, opioid use, and pain, showed no significant differences between TIVA and inhalational anesthesia in the present analysis.

**Conclusion:**

TIVA appears to improve perioperative outcomes in obese patients by reducing PONV and intraoperative heart rate, highlighting its potential advantages in clinical practice. Further research is needed to address variability and establish evidence-based guidelines for anesthesia management in this high-risk population.

**Systematic review registration Number in PROSPERO:**

CRD42024547776

**Supplementary Information:**

The online version contains supplementary material available at 10.1186/s44158-025-00234-1.

## Introduction

Obesity is a global epidemic projected to affect 4 billion people by 2035 [1]. Characterized by excess body fat and a body mass index (BMI) greater than 30 kg/m^2^, it significantly increases the risk of mortality and noncommunicable diseases [2, 3]. In the USA, nearly 75% of adults aged 20 years and older are overweight (BMI > 25 kg/m^2^) or obese [4, 5]. Globally, high BMI contributed to an estimated 5 million deaths in 2019, and the economic burden of obesity is projected to reach $4.3 trillion annually by 2035 if effective interventions are not implemented [6]. This growing prevalence poses a significant challenge in perioperative care, particularly for anesthesiologists managing patients with obesity, where general anesthesia is frequently used to ensure effective pain control and hemodynamic stability in this high-risk population [7]. However, anesthesia regimens present distinct challenges in this population. Volatile anesthetics are commonly associated with postoperative nausea and vomiting (PONV), while total intravenous anesthesia (TIVA) carries risks such as potential hyperalgesia and higher costs [7]. Recent evidence suggests that TIVA may reduce PONV, facilitate faster discharge, and improve cognitive recovery compared to inhalation anesthesia. However, its effectiveness in obese patients remains unclear, particularly regarding how factors such as surgery type, choice of inhalational agent, or variables like heart rate (HR) and mean arterial pressure (MAP) influence outcomes [8]. This systematic review and meta-analysis aims to evaluate the safety and efficacy of inhalation anesthesia versus TIVA in obese patients undergoing surgery. Our primary outcomes include postoperative nausea and vomiting (PONV), recovery time, postoperative heart rate (HR), and mean arterial pressure (MAP). Secondary outcomes include postoperative pH, ICU length of stay, and oxygen saturation (SpO2) at the end of surgery.

## Methods

The present systematic review followed the recommendations and criteria established by the Preferred Reporting Items for Systematic Reviews and Meta-analyses (PRISMA) reporting guidelines [9]. The protocol was preregistered at the International Prospective Register of Systematic Reviews with the identifier code CRD42024547776. We included in the meta-analysis adult patients (aged 18 or older) affected by obesity (BMI ≥ 30 kg/m^2^), undergoing general anesthesia (P) and receiving an inhalation agent (I) or TIVA (C). The primary outcomes included PONV, recovery time, and postoperative HR/MAP. Secondary outcomes included postoperative pH, ICU length of stay, and SpO2 at the end of the surgery (O), including only randomized controlled trials (RCT) written in English or Spanish (S).

### Search methods

A systematic search was conducted in PubMed MEDLINE, Web of Science, Scopus, China National Knowledge Infrastructure, Cumulative Index to Nursing and Allied Health Literature, Cochrane, EMBASE, and Google Scholar (retrieving the first 100 results) using the following terms: obesity, perioperative outcomes, and general anesthesia. All detailed search strategies can be found in the supplementary material (Supplementary Tables 1–8).

### Selection of studies

All references were exported to Rayyan (Rayyan Systems Inc., Cambridge, MA, USA) [10], and duplicates were removed. Two authors independently (AT and LCR) completed the eligibility assessment, first by title and abstract analysis and, subsequently, by full-text assessment. In disagreements between reviewers, a third reviewer (SSD) helped reach a consensus.

### Data extraction

Two independent reviewers extracted the data (JNL and MDTC), and disagreements were resolved by consensus. When multiple overlapping reports from the same study were identified, the information from the one containing the most relevant information or the first published report was included. Extracted data included sample sizes, intervention types, and measured outcomes. Conventional methods were used for data extraction, complemented by specialized tools such as WebPlotDigitizer (Automeris, Austin, TX, USA) for digitizing data from graphs, Cochrane Calculator for deriving statistical measures from available data, and StatsToDo for advanced calculations [11–13]. These outcomes comprised the incidence of postoperative nausea and vomiting, time to emergence from anesthesia, postoperative pH values, ICU stay duration, intraoperative HR, MAP, and morphine usage. All variables were extracted from data measured postoperatively. Recovery time was defined as the time to the earliest documented recovery event, prioritizing eye-opening, followed by awakening, and then name stating. PONV, HR, and MAP were extracted when measured in the postoperative period, with HR and MAP taken from the first documented value after surgery, prioritizing values within the first postoperative hour. Pain was assessed primarily during the immediate postoperative period or the first documented report postoperatively. Morphine requirements were extracted when measured at 24–48 h postoperatively. Additional extracted data included subgroup characteristics, such as country of study, risk of bias levels, obesity classifications, and types of surgeries performed.

### Assessment of risk of bias in included studies

To assess the quality of the studies included in the systematic review, according to the Cochrane guidelines, we applied the Cochrane RoB 2.0 tool for randomized controlled trials (RCTs) [14–16]. Two independent reviewers (VPSV, MMTC) evaluated the risk of bias in each study, considering the specific criteria and guidelines provided by the respective tools. Any reviewer discrepancies were resolved through discussion with a third, blinded reviewer (SSD).

### Statistical analysis

A meta-analysis was performed using R version 3.4.3 (R Core Team) with the meta and metafor packages [17, 18]. The pooled effect of the outcomes was examined using a random-effects meta-analysis (DerSimonian-Laird approach) [19]. Whenever the number of studies reporting an outcome of interest was insufficient, only a qualitative analysis of the results was performed. Effect sizes were expressed as relative risk (RR), mean difference (MD), or standardized mean difference (SMD) with a 95% confidence interval. The *I*^2^ statistic assessed heterogeneity, and the following cut-off values were used for interpretation: < 25%, 25–50%, and > 50% were considered small, medium, and large heterogeneity, respectively [14]. For all outcomes, sensitivity analyses using the leave-one-out method were performed to determine the influence of individual studies on the overall effect [20]. Egger’s regression test was used to examine publication bias when 10 or more reports with the same outcome were available [21]. Whenever possible, subgroup analyses were planned based on risk of bias, inhalational agent, type of surgery, and ASA level for the specified outcomes.

## Results

### Study selection

**Fig. 1:**
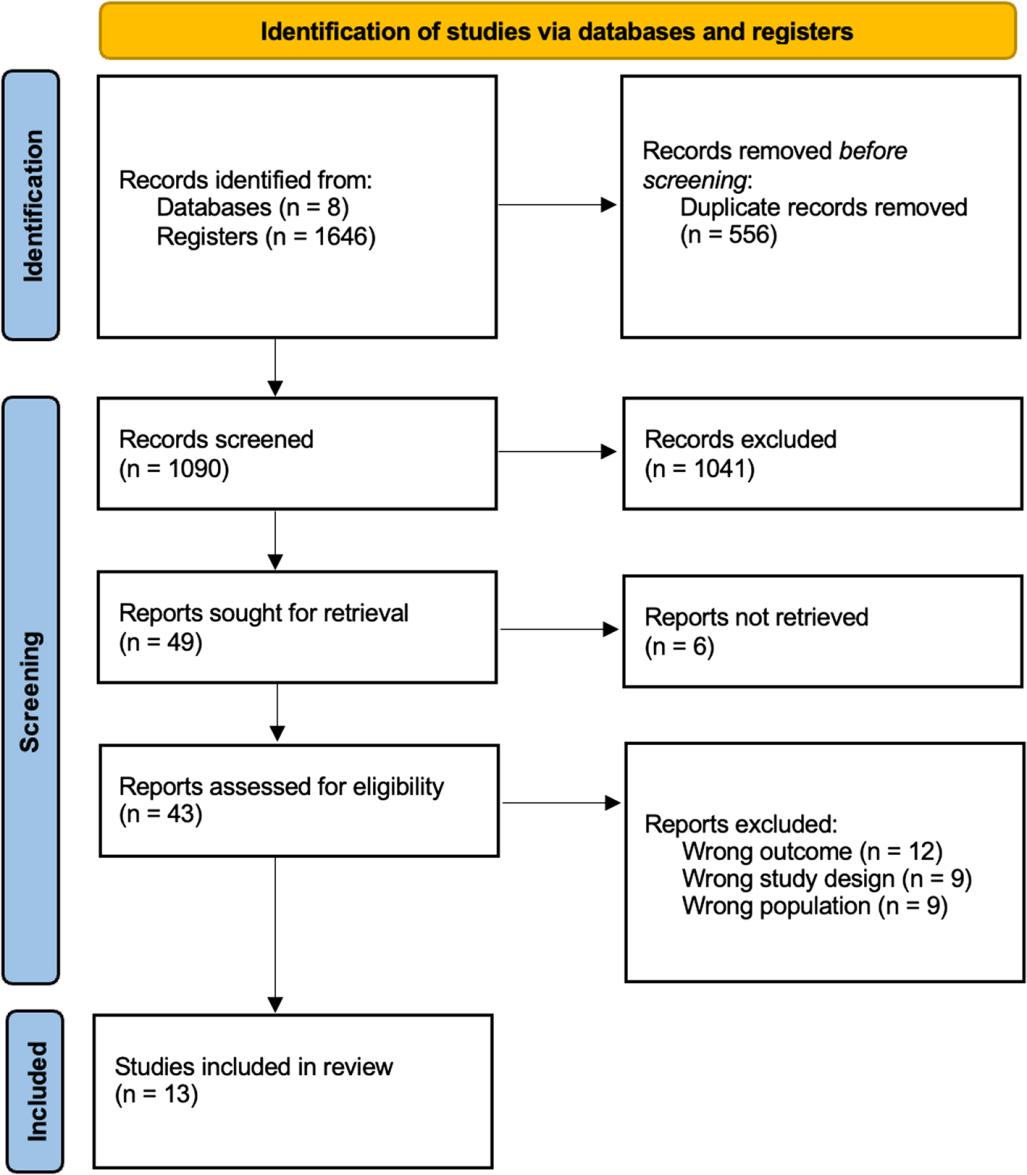
PRISMA flow diagram. Prisma flow diagram delineates the systematic process of identifying and screening studies across multiple databases, culminating in selecting 13 pertinent studies

Our search performed on 01/15/2025 identified 1646 possible articles across eight different databases. After removing 556 duplicate articles, we screened the titles and abstracts of the remaining articles, excluding 1041 articles. Subsequently, 49 publications were sought for retrieval, and 6 were further removed from the screening process. The remaining publications were assessed for eligibility. Out of the 43 articles, 29 were excluded due to an incorrect outcome (12), an incorrect population (9), or an incorrect study design (9). Ultimately, 13 publications were assessed and included in the final review process. These findings are summarized in our PRISMA flow chart (Fig. 1).

### Characteristics of the included studies

The total sample size of the thirteen publications was 1072 participants. These studies were conducted in various geographic locations, including Turkey, the United States of America, Norway, Egypt, Greece, Indonesia, France, and China. The primary findings from these randomized controlled trials (RCTs) focused on investigating the effects of different anesthetic agents in patients with obesity during elective surgery. Eleven of the thirteen studies focused on bariatric surgery, one on minor peripheral surgery, and one on total knee arthroplasty. PONV data were available in seven studies. Three studies reported significantly higher incidences of PONV in the inhalational group, and four studies found no significant differences. Anesthesia recovery time was discussed in seven studies, with four reporting significantly shorter recovery times in the TIVA group, two studies reporting no significant differences, and one study finding early recovery with desflurane. The remaining studies did not provide data on PONV or recovery time. Seven studies assessed intraoperative or postoperative vital signs, specifically HR and MAP. One study reported significantly lower intraoperative HR in the TIVA group, while six others found no significant differences in HR. Regarding MAP, two studies reported significantly lower intraoperative or postoperative MAP in the TIVA group, and four studies found no significant differences in between groups. One study observed lower postoperative MAP in the sevoflurane group. Seven studies reported pain, with six of them finding no significant differences postoperatively. Among these, one study (Shu et al.) assessed pain at 24 h, while the majority of the remaining studies evaluated pain within the first 3 h after surgery. One of these studies reported differences at 6–8 h in the TIVA group. One article reported reduced pain scores in the propofol group. Regarding morphine requirements, two articles report lower requirements in the TIVA group, and three articles have no differences. These findings are summarized in the general outcome table (Table 1) [22–34].

**Table 1:** General outcomes

**Author**	**Year**	**Country**	**Intervention**	**Comparator**	**Sample size (total)**	**BMI** patients mean (SD)	**ASA classification**	**Surgery type**	**Key points**
Babayigit et al. [22]	2020	Turkey	Sevoflurane	Propofol	55	S, 45.19 (3.78) P, 46.45 (4.77)	I, II, or III	Bariatric	Demographic data, MAP, and HR were similar between groups. IOP decreased significantly after anesthesia induction in both groups with no significant difference between groups. Sevoflurane and TIVA demonstrated similar effects on IOP
Demirel et al. [23]	2017	Turkey	Desflurane	Propofol	120	P, 45.47 (3.90) D, 47.8 (12.3)	NA	Bariatric	Demographic data, anesthesia duration, and perioperative MAP and HR were similar between groups. Recovery time was significantly shorter, and nausea and vomiting were significantly lower in the TIVA group
Elbakry et al. [24]	2018	Egypt	Desflurane	Propofol	100	D, 42.55 (4.36) P, 41.60 (4.38)	III	Bariatric	Recovery time was significantly shorter in the TIVA group. Nausea and vomiting were significantly more frequent in the desflurane group. TIVA group had lower intraoperative MAP, HR, and pain scores. Morphine use was higher in the desflurane group
Juvin et al. [25]	2000	France	Desflurane	Propofol	23	D, 46.5 (5.3) P, 47.5 (4.9)	NA	Bariatric	No significant differences in PONV or pain between groups. PACU time was shorter in the desflurane group. No differences in morphine requirements were observed
Lehavi et al. [26]	2015	USA	Sevoflurane	Propofol	30	I, 43 (4) P, 43 (2)	III or IV	Bariatric	No significant differences in CPK or troponin I levels between groups. No cases of rhabdomyolysis were reported. pH levels were significantly lower in the inhalational agent group
Salighoglu et al. [27]	2001	Turkey	Sevoflurane	Propofol	40	50 (35–64)	I or II	Bariatric	MAP was significantly reduced in the TIVA group after surgery. No significant differences were found in HR, pH, SpO2, or eye-opening times after surgery
Tanaka et al. [28]	2017	USA	Desflurane	Propofol	90	D, 36.5 (35.0–38.1) P, 34.0 (33.0–35.0)	II or III	Knee arthroplasty	Pain scores at 6–8 h were significantly lower with propofol. No significant differences in PONV, morphine use, or wake-up times. Cognitive function decreased in 50% of patients on the first 2 postoperative days
Ziemann et al. [29]	2014	USA	Sevoflurane/Desflurane	Propofol	159	S/D, 45.32 (6.97) P, 44.15 (7.46)	NA	Bariatric	PONV incidence was significantly lower in the TIVA group (20%) compared to the inhalant group (37.3%). No significant differences were found in opioid consumption, pain scores, or PACU time between groups
Honca et al. [30]	2017	Turkey	Desflurane	Propofol	61	I, 44.5 (4.59) P, 43.5 (4.46)	I or II	Bariatric	No significant differences in PONV incidence among groups. Time to eye-opening was significantly shorter in the sevoflurane group. No significant differences in postoperative MAP or HR
Mutholib et al. [31]	2024	Indonesia	Sevoflurane/Desflurane	Propofol	80	> 40 kg/m2	I, II, or III	Multiple (bariatric (37.5%, orthopedic (31.3%) Urolgy (18.85) gynecology (12.5%)	Recovery time was significantly shorter in the TIVA group. Opioid requirements were lower in the TIVA group compared to the inhalation group
Shu et al. [32]	2024		Sevoflurane	Propofol		S: 37.09 (1.64) P:36.88 (1.18)	II or III	Bariatric	Demographic data, anesthesia duration, and recovery times were similar between groups. The propofol group had no differences in postoperative MAP and HR compared to the sevoflurane group. PONV incidence was equal into the groups. No differences found in PACU time No differences for pain assessment
Aftab et al. [33]	2019	Norway	Desflurane	Propofol	183	D: 43 (6.0) P:43 (6.0)	I, II, or III	Bariatric	No significant differences were found in nausea, pain, or wake-up times. PONV was significantly more frequent in the desflurane group at 1–3 and 6–24 h postoperatively. Morphine requirements were not different among groups
Siampalioti et al. [34]	2015	Greece	Sevoflurane	Propofol	100	S, 59 (9.6) P, 57 (8.9)	NA	Bariatric	Time to eye-opening and extubation was significantly shorter in the propofol group. No significant differences in postoperative pain scores or HR. MAP was significantly lower in the sevoflurane group postoperatively

*MAP* mean arterial pressure, *PACU* Post-Anesthesia Care Unit, *TIVA* total intravenous anesthesia, *CPK* creatine phosphokinase, *BP* blood pressure, *HR* heart rate, *IOP* intraocular pressure, *PONV* postoperative nausea and vomiting, *VAS* Visual Analogue Scale, *SpO2* peripheral capillary oxygen saturation,* BIS* Bispectral Index, *D* desflurane, *S* sevoflurane, *P* propofol, *I* isoflurane.

### Risk of bias

**Fig. 2:**
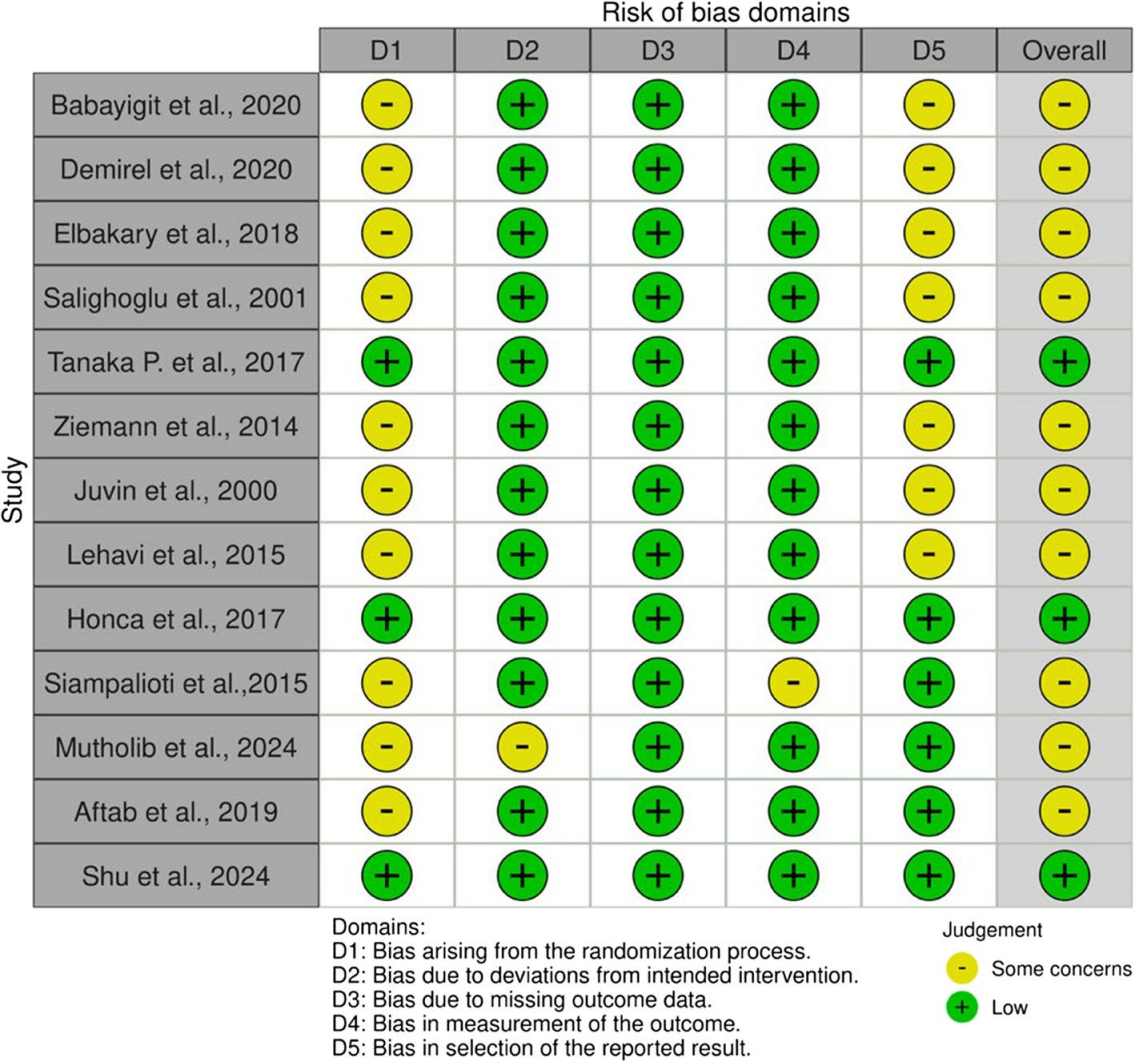
Risk of bias

From the fourteen articles included, 10 articles (77%) presented some concerns about bias, and three (23%) showed a low risk of bias (Fig. 2). The overall results are categorized into two colors: yellow for some concerns and green for low risk. Our selection showed that all the publications resulted in a low risk or some concerns, with none categorized as high risk.

### Meta-analysis

#### Postoperative vomiting and nausea

**Fig. 3:**
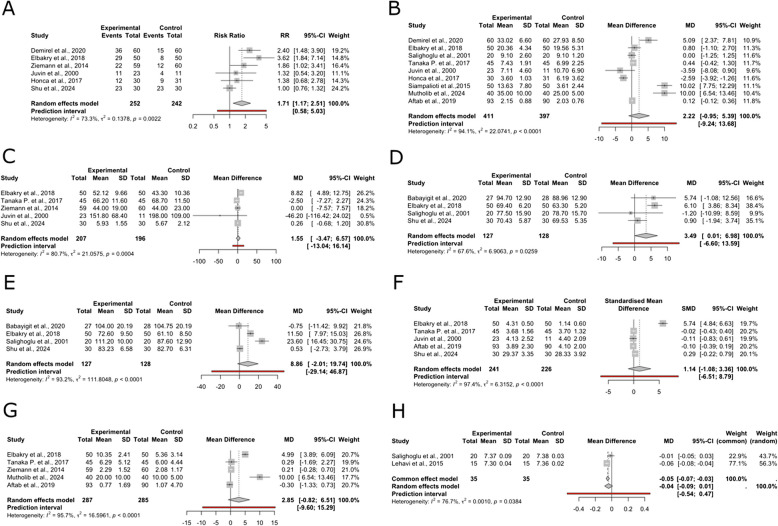
Forest plots. **A** Vomiting and nausea. **B** Time to emergence. **C** ICU stay duration. **D** Heart rate. **E** MAP. **F** Pain. **G** Morphine. **H** Postoperative pH

The meta-analysis for PONV included six studies with 494 observations. The random-effects model yielded an RR of 1.71 (95% CI, 1.16 to 2.51; *p* < 0.01, *I*^2^ = 73.3%) under inhalational anesthesia (Fig. 3A). The funnel plot shows asymmetry, suggesting potential publication bias or small-study effects. Egger’s test was not feasible due to the low number of studies (Supplementary Fig. 1).

### Subgroup and sensitivity analysis

Subgroup analysis revealed statistically significant differences based on the inhalational agent (*p* = 0.01) and ASA classification of patients (*p* < 0.01). Patients receiving desflurane showed reduced heterogeneity and a significant RR of 2.09 (95% CI, 1.33 to 3.29; *I*^2^ = 42.1%). Studies that did not report ASA levels exhibited no heterogeneity and a significant RR of 2.01 (95% CI, 1.42 to 2.85; *I*^2^ = 0.0%). Sensitivity analysis identified Demirel et al. (2020), Elbakry et al. (2018), and Shu et al. (2024) as influential studies (Supplementary Table 10). Excluding these studies reduced heterogeneity to zero and yielded a significant RR of 1.56 (95% CI, 1.04 to 3.35; *p* = 0.03; *I*^2^ = 0.0%). No other significant results were found in the meta-analysis or subgroup analyses for this outcome.

### Time to emergence

Nine studies with 808 observations were included in the analysis of the time to emergence from anesthesia. The random-effects model produced a MD of 2.21 (95% CI, − 0.94 to 5.38; *p* = 0.16; *I*^2^ = 94.1%) under inhalational anesthesia (Fig. 3B). The funnel plot shows asymmetry, suggesting potential publication bias or small-study effects. Egger’s test was not feasible due to the low number of studies (Supplementary Fig. 1).

### Subgroup and sensitivity analysis

Subgroup analysis revealed statistically significant differences based on the inhalational agent (*p* < 0.01), risk of bias (*p* < 0.01), and type of surgery (*p* < 0.01). Only the analysis of low-risk-of-bias studies resulted in a reduction in heterogeneity. None of the subgroups for the type of surgery, risk of bias, or inhalational agent led to a change in the effect size (Supplementary Table 11). Sensitivity analysis identified Honca et al. (2017), Siampalioti et al. (2015), and Aftab et al. (2019) as influential studies (Supplemental Table 12). An analysis excluding these studies resulted in a non-significant MD of 2.11 (95% CI, − 1.41 to 5.65; *p* = 0.24; *I*^2^ = 88.2%). No other significant results were found in the meta-analysis or subgroup analyses for this outcome.

### ICU stay duration

The analysis of ICU duration included four studies with 403 observations. The random-effects model showed a MD of 1.54 (95% CI, − 3.47 to 6.57; *p* = 0.54; *I*^2^ = 80.7%) under inhalational anesthesia (Fig. 3C). The funnel plot shows asymmetry, suggesting potential publication bias or small-study effects. Egger’s test was not feasible due to the low number of studies (Supplementary Fig. 1).

### Subgroup and sensitivity analysis

Subgroup analysis revealed statistically significant differences based on ASA classification (*p* < 0.01). Only the subgroup of ASA classification III, based on one study, yielded a significant MD of 8.82 (95% CI, 4.89 to 12.74; Supplementary Table 13). Sensitivity analysis identified Elbakry et al. (2018) and Shu et al. (2024) as influential studies (Supplementary Table 14). Excluding these studies resulted in a non-significant MD of − 1.93 (95% CI, − 5.96 to 2.09; *p* = 0.34; *I*^2^ = 0.0%). No other significant results were found in the meta-analysis or subgroup analyses for this outcome.

### Heart rate

The analysis of first HR record after surgery included four studies with 255 observations. The random-effects model showed a MD of 3.49 (95% CI, 0.01 to 6.97; *p* < 0.01; *I*^2^ = 67.6%) under inhalational anesthesia (Fig. 3D). The funnel plot shows asymmetry, suggesting potential publication bias or small-study effects. Egger’s test was not feasible due to the low number of studies (Supplementary Fig. 1).

### Subgroup and sensitivity analysis

Subgroup analysis revealed statistically significant differences based on ASA classification (*p* = 0.02) and inhalational agent (*p* < 0.01). The subgroup of sevoflurane showed decreased heterogeneity with a non-significant MD of 1.42 (95% CI, − 1.10 to 3.96; *I*^2^ = 0.0%). Subgroups for ASA classification were based on single-article groups (Supplementary Table 15). Sensitivity analysis identified Elbakry et al. (2018) and Shu et al. (2024) as influential studies (Supplementary Table 16). Excluding these studies resulted in a non-significant MD of 3.19 (95% CI, − 3.35 to 9.75; *p* = 0.33, *I*^2^ = 23%). No other significant results were found in the meta-analysis or subgroup analyses for this outcome.

#### MAP

The analysis of the first MAP record after surgery included four studies with 255 observations. The random-effects model showed a MD of 8.86 (95% CI, − 2.01 to 19.73; *p* = 0.11, *I*^2^ = 93.2%) under inhalational anesthesia (Fig. 3E). The funnel plot shows asymmetry, suggesting potential publication bias or small-study effects. Egger’s test was not feasible due to the low number of studies (Supplementary Fig. 1).

### Subgroup and sensitivity analysis

Subgroup analysis revealed statistically significant differences based on ASA classification (*p* < 0.01). ASA subgroups were based on single-article groups (Supplementary Table 17). Sensitivity analysis identified Elbakry et al. (2018), Salighoghlu et al. (2001), and Shu et al. (2024) as influential studies (Supplementary Table 18). An analysis excluding three of the four identified studies could not be carried out due to data limitations. No other significant results were found in the meta-analysis or subgroup analyses for this outcome.

### Pain

The analysis of pain after surgery included five studies with 467 observations. The random-effects model showed an SMD of 1.14 (95% CI, − 1.08 to 3.36; *p* = 0.31, *I*^2^ = 97.4%), under inhalational anesthesia (Fig. 3F). The funnel plot suggests asymmetry, potentially indicating publication bias or small-study effects. Notably, one study with a high SMD appears to drive this asymmetry. Egger’s test was not feasible due to the low number of studies (Supplementary Fig. 1).

### Subgroup and sensitivity analysis

Subgroup analysis revealed statistically significant differences based on ASA classification (*p* < 0.01). ASA subgroups were based on single-article groups (Supplementary Table 19). Sensitivity analysis identified Elbakry et al. (2018), Salighoghlu et al. (2001), and Shu et al. (2024) as influential studies (Supplementary Table 20). Notably, excluding Elbakry et al. (2018) led to a decrease of heterogeneity with a SMD of − 0.01 (95% CI, − 0.22 to 0.19; *p* = 0.88, *I*^2^ = 0.0%). An analysis excluding three of the four identified studies could not be carried out due to data limitations. No other significant results were found in the meta-analysis or subgroup analyses for this outcome.

### Morphine

The analysis of morphine after intervention included five studies with 572 observations. The random-effects model showed a MD of 2.85 (95% CI, − 0.82 to 6.51; *p* = 0.12; *I*^2^ = 95.7%), under inhalational anesthesia (Fig. 3G). The funnel plot shows asymmetry, suggesting potential publication bias or small-study effects. Egger’s test was not feasible due to the low number of studies (Supplementary Fig. 1).

### Subgroup and sensitivity analysis

Subgroup analysis revealed statistically significant differences based on ASA classification (*p* < 0.01), bias risk (*p* < 0.01), and surgery type (*p* < 0.01). However, the differences among all subgroups were based on single-article groups (Supplementary Table 21). Subgroup analysis by inhalational agent did not reveal any significant differences. Sensitivity analysis identified Elbakry et al. (2018), Ziemann et al. (2014), and Aftab et al. (2019) as influential studies (Supplementary Table 22). Excluding these studies resulted in a non-significant mean difference (MD) of 5.03 (95% CI, − 4.47 to 14.45; *p* = 0.29; *I*^2^ = 95.6%). No other significant results were found in the meta-analysis or subgroup analyses for this outcome.

### Postoperative pH values

The analysis of postoperative pH values included two studies with 70 observations. The random-effects model yielded a MD of − 0.03 (95% CI, − 0.08 to 0.01; *p* = 0.12; *I*^2^ = 76.7%) under inhalational anesthesia (Fig. 3H). The funnel plot shows asymmetry, suggesting potential publication bias or small-study effects. Egger’s test was not feasible due to the low number of studies (Supplementary Fig. 1). Subgroup and sensitivity analysis were not possible due to the low number of included studies.

### Grading of recommendations, assessment, development, and evaluation assessment

A Grading of Recommendations, Assessment, Development, and Evaluation approach was applied to assess all variables. The analysis revealed a low level of certainty across all variables (Supplementary Table 23).

## Discussion

This systematic review and meta-analysis provides a comprehensive comparison of inhalation anesthesia and TIVA on perioperative complications in patients with obesity, representing the first study to evaluate vital signs with a larger sample size than previously done. This study addresses several methodological limitations of earlier reviews by strictly adhering to PRISMA guidelines, employing dual independent data extraction, and conducting rigorous quality assessments of included studies. These advancements strengthen the evidence guiding anesthetic regimens in obese patients, aiming to enhance perioperative safety and improve outcomes in this high-risk population.

In our analysis, the incidence of PONV was approximately two times greater under inhalational anesthesia than TIVA, with an RR of 1.71 (95% CI, 1.16 to 2.51; *p* < 0.01, *I*^2^ = 73.3%), being more evident in the desflurane subgroup [35]. This finding aligns with previous studies, such as Ahmed et al. who reported a significantly reduced incidence of nausea and vomiting in the TIVA group by 46% and 69%, respectively [36]. However, Ahmed et al.’s analysis was limited by methodological flaws, including double-counting, which overstates results [37]. Our updated analysis avoids these errors and offers a larger, more robust dataset. Additionally, findings from a second meta-analysis supported higher rates of PONV with inhalational agents, particularly desflurane, in patients with obesity, further corroborating our results. [38]. The clinical implications of elevated PONV rates in obese patients are significant, as they are at increased risk for complications such as postoperative atelectasis and hypoxia [39]. Given the strong association between inhalational anesthetics and PONV, targeted prophylactic strategies are essential in this population. Evidence suggests that a multimodal antiemetic approach, including the use of pharmacologic agents with different mechanisms of action, can mitigate PONV risk in high-risk patients [40]. Specifically, the administration of dexamethasone, ondansetron, or newer agents such as palonosetron or aprepitant has been shown to be effective in reducing postoperative nausea and vomiting [41]. Furthermore, opioid-sparing analgesic techniques, including regional anesthesia and non-opioid adjuncts, may contribute to improved PONV control by reducing reliance on emetogenic opioids [41]. For obese patients undergoing high-risk surgeries (e.g., intra-abdominal, gynecological, or maxillofacial procedures), TIVA may provide a safer anesthetic option [5], particularly when combined with comprehensive prophylactic measures against PONV.

Postoperatively, MAP and HR values demonstrated high heterogeneity, which persisted despite subgroup analyses, limiting the robustness of inferences. This heterogeneity likely arises from differences in the timing of measurements, although all data were recorded within the first postoperative hour. The meta-analysis revealed a statistically significant increase in HR with inhalational anesthesia (MD 3.49 beats per minute); however, the clinical relevance of this finding is minimal. MAP results, though not statistically significant, exhibited a tendency to increase with inhalational agents. These findings are clinically relevant given the heightened sympathetic activity and vagal withdrawal observed in obese patients which may exacerbate hemodynamic instability during anesthesia [42, 43]. For example, inhalational agents such as desflurane induce tachycardia and hypertension through sympatho-excitation and vagolytic effects, as evidenced by increased sympathetic nerve activity [42, 44]. This highlights the need for caution when using desflurane, particularly in high-risk populations with obesity or cardiovascular comorbidities.

While no statistical significant differences were observed between inhalational anesthesia and TIVA in recovery time, consistent with Lin et al.’s findings based on two studies, the limited sample size in previous analyses reduces generalizability [45]. In contrast, our study includes a larger sample size, providing more reliable conclusions.

Recovery time was not statistically significant in our analysis, and this contains differences with prior analysis performed for specific agents such as desflurane, sevoflurane, and propofol, but these were affected by small sample sizes and methodological issues, including double-counting [38, 46]. For example, Hu Z et al. favored propofol for extubation times but was limited by double-counting mistakes [46]. Our analysis addresses these limitations, offering improved generalizability and minimizing bias. No significant differences were found in postoperative ICU duration, pH values, pain, or morphine requirements between anesthetic techniques. This aligns with Ahmed et al.’s findings but highlights the need for further research to confirm these results due to small subgroup sizes [36]. Although ASA classification subgroup analysis suggested higher pain scores and morphine use in ASA III patients, these findings are derived from single-study subgroups and should be interpreted cautiously. While this underscores the necessity of optimizing perioperative analgesia irrespective of anesthetic technique, the limited data precludes definitive conclusions regarding the efficacy of opioid-sparing strategies in this population [47]. Nonetheless, the incorporation of multimodal analgesia, including adjunctive non-opioid pharmacologic agents and regional anesthesia, may contribute to enhanced pain control and mitigation of opioid-related adverse effects in ASA III patients [48].

This study emphasizes the importance of tailoring anesthetic regimens to the unique needs of obese patients. TIVA demonstrated significant reductions in intraoperative HR and PONV incidence, which is critical for minimizing perioperative risks and enhancing recovery. However, the variability in MAP and recovery times highlights gaps in the current knowledge, warranting further research to refine anesthesia strategies and improve outcomes in this high-risk population.

## Limitations

Our study has several limitations that must be considered when interpreting the results. The primary limitation is the limited number of studies available for analysis, as we included only thirteen randomized controlled trials (RCTs). Non-English or Spanish publications may have been omitted, which could introduce publication bias and limit the diversity of perspectives considered. Future studies should aim to include a broader range of languages to minimize this bias and capture a more global perspective. Additionally, differences in anesthetic administration regimens, concomitant drugs such as opioids or alpha-2 agonists, dosages, perioperative care, and specific timing of the vital signs could impact patient outcomes and limit our ability to draw firm conclusions. Another limitation is the variability in how recovery time is measured across studies. Recovery time is often defined using different criteria, such as eye-opening, awakening, or stating one’s name, which makes it difficult to standardize outcomes and compare results across studies. Future research should adopt uniform definitions and standardized protocols for anesthetic regimens of recovery time to enhance consistency and facilitate meta-analyses. The lack of long-term follow-up restricts our understanding of any delayed effects or complications that may arise after discharge. Studies with extended follow-up periods are needed to evaluate the long-term safety and efficacy of anesthetic strategies in obese patients. Moreover, incomplete data reporting in some studies could lead to biases and incomplete data synthesis. Potential confounding factors, such as variations in surgical techniques, perioperative care, and patient characteristics, may also influence the outcomes. Future investigations should control for these factors through rigorous study design and statistical analyses to provide clearer insights. By addressing these limitations, future studies could establish more definitive conclusions and better guide perioperative care to reduce complications and improve outcomes for obese patients undergoing surgery.

## Conclusion

This systematic review (PROSPERO (CRD42024547776)) and meta-analysis provide valuable insights into anesthetic strategies for obese patients, emphasizing the need for a tailored, evidence-based approach to optimize perioperative outcomes. The findings demonstrate that TIVA significantly reduces the risk of PONV and lowers intraoperative HR, offering potential benefits in enhancing patient safety and comfort. However, no significant differences were observed in MAP, ICU length of stay, recovery time, pH, pain, or opioid use, underscoring the need to address these outcomes through further research. The variability across studies highlights critical gaps in evidence, particularly regarding MAP and recovery time, which remain inconclusive due to methodological differences and limited sample sizes. Future research should focus on standardizing outcome definitions, evaluating long-term effects, and exploring patient-specific factors to guide anesthetic choices. By addressing these challenges, clinical practice can be improved to reduce complications and enhance the quality of care for this high-risk population.

## Supplementary Information

Additional file 1.

## Data Availability

No datasets were generated or analysed during the current study.

## Abbreviations

WHO: The World Health Organization
BMI: Body mass index
TIVA: Total intravenous anesthesia
PRISMA: Preferred Reporting Items for Systematic Reviews and Meta-Analyses
PROSPERO: International Prospective Register of Systematic Review
CNKI: China National Knowledge Infrastructure
CINDAHL: Cumulative Index to Nursing and Allied Health Literature
ICU: Intensive care unit
RCTs: Randomized controlled trials
OR: Odds ratio
CI: Confidence interval
MD: Mean differences
SMD: Standardized mean difference
NCDs: Non-communicable diseases
GA: General anesthesia
PACU: Post anesthesia care unit
RoB 2.0: Risk of Bias 2.0
CPK: Creatine phosphokinase
MAP: Mean arterial pressure
HR: Heart rate
NIBP: Noninvasive blood pressure
SpO2: Peripheral capillary oxygen saturation
