# Supplementary material for: Inhalation anesthesia and total intravenous anesthesia (TIVA) regimens in patients with obesity: an updated systematic review and meta-analysis of randomized controlled trials
Source: J Anesth Analg Crit Care. 2025 Mar 18;5:15. doi: 10.1186/s44158-025-00234-1 (PMC11917146; doi:10.1186/s44158-025-00234-1)
Supplement: Supplementary file 1 — Additional file 1. [file 44158_2025_234_MOESM1_ESM.docx]

**Supplementary Table 1. PubMed Search Results**

| **Search** | **Results** |
| --- | --- |
| ((intravenous anesthesia[MeSH Terms]) OR (Intrevenous anesthesia[Title/Abstract]) OR (TIVA[Title/Abstract]) OR (Total Intravenous Anesthesia[Title/Abstract]) OR (Total Intravenous Anes*[Title/Abstract]) OR (fentanyl[Title/Abstract]) OR (sufentanil[Title/Abstract]) OR (remifentanil[Title/Abstract]) OR (iv anesthesia[Title/Abstract]) OR (propofol[Title/Abstract]) OR (anaesthetics intravenous[Title/Abstract])) AND ((inhalation anesthesia[MeSH Terms]) OR (inhalation anaesthetics[MeSH Terms]) OR (inhalation anesthesia[Title/Abstract]) OR (inhalation anaesthetics[Title/Abstract]) OR (Volatile Anesthesia[Title/Abstract]) OR (desflurane[Title/Abstract]) OR (sevoflurane[Title/Abstract]) OR (isoflurane[Title/Abstract]) OR (volatile anaesthetics[Title/Abstract]) OR (inhalational anesthesia[Title/Abstract]) OR (inhaled anesthesia[Title/Abstract])) AND ((obesity[MeSH Terms]) OR (morbid obesities[MeSH Terms]) OR (obesity[Title/Abstract]) OR (morbid obesities[Title/Abstract]) OR (Obese[Title/Abstract]) OR (Severely Obese[Title/Abstract]) OR (overweight[Title/Abstract])) | 138 results |

**Supplementary Table 2. Web of Science Search Results**

| **Query Number** | **Search Term** | **Results Count** |
| --- | --- | --- |
| 1 | All=Anesthesia, Intravenous | 24,990 |
| 2 | All=TIVA | 1,707 |
| 3 | All=Total Intravenous Anesthesia | 7,304 |
| 4 | All=Total Intravenous Anes* | 9,999 |
| 5 | All=Fentanyl | 27,877 |
| 6 | All=Sufentanil | 4,912 |
| 7 | All=Remifentanil | 8,753 |
| 8 | All=IV Anesthesia | 9,893 |
| 9 | All=Propofol | 35,764 |
| 10 | All=Anaesthetics Intravenous | 12,881 |
| 11 | (#10 OR #9 OR #8 OR #7 OR #6 OR #5 OR #4 OR #3 OR #2 OR #1) | 91,264 |
| 12 | All=Anesthesia, Inhalation | 5,203 |
| 13 | All=Anesthetics, Inhalation | 4,079 |
| 14 | All=Inhalation Anesthesia | 5,203 |
| 15 | All=Volatile Anesthesia | 5,440 |
| 16 | All=Desflurane | 4,378 |
| 17 | All=Sevoflurane | 15,609 |
| 18 | All=Isoflurane | 21,803 |
| 19 | All=Volatile Anaesthetics | 8,883 |
| 20 | All=Inhalational Anesthesia | 2,152 |
| 21 | All=Inhaled Anesthesia | 3,375 |
| 22 | (#12 OR #13 OR #14 OR #15 OR #16 OR #17 OR #18 OR #19 OR #20 OR #21) | 42,296 |
| 23 | All=Obesity | 567,861 |
| 24 | All=Overweight | 144,767 |
| 25 | All=Obese | 207,930 |
| 26 | (#23 OR #24 OR #25) | 674,902 |
| 27 | (#26 AND #22 AND #11) | 198 |

**Supplementary Table 3. Scopus Search Results**

| **Search** | **Results** |
| --- | --- |
| intravenous AND anesthesia AND volatile AND anesthesia AND obesity | 27 results |

**Supplementary Table 4. CNKI Search Results**

| **Search** | **Results** |
| --- | --- |
| ((SU=(intravenous anesthesia) OR SU=(Intrevenous anesthesia) OR SU=(TIVA) OR SU=("Total Intravenous Anesthesia") OR SU=("Total Intravenous Anes*") OR SU=("iv anesthesia")) AND  (SU=("inhalation anesthesia") OR SU=("inhalation anaesthetics") OR SU=("Volatile Anesthesia") OR SU=("volatile anaesthetics") OR SU=("inhalational anesthesia") OR SU=("inhaled anesthesia")) AND (SU=(obesity) OR SU=("morbid obesities") OR SU=(Obese) OR SU=("Severely Obese") OR SU=(overweight))) | 204 results |

**Supplementary Table 5. CINDAHL Search Results**

| **Search** | **Results** |
| --- | --- |
| ( (MH "Intravenous Anesthesia" OR TI "Intravenous anesthesia" OR AB "Intravenous anesthesia" OR TI TIVA OR AB TIVA OR TI "Total Intravenous Anesthesia" OR AB "Total Intravenous Anesthesia" OR TI "Total Intravenous Anes*" OR AB "Total Intravenous Anes*" OR TI Fentanyl OR AB Fentanyl OR TI Sufentanil OR AB Sufentanil OR TI Remifentanil OR AB Remifentanil OR TI "IV Anesthesia" OR AB "IV Anesthesia" OR TI Propofol OR AB Propofol OR TI "Anaesthetics Intravenous" OR AB "Anaesthetics Intravenous") ) AND ( (MH "Inhalation Anesthesia" OR TI "Inhalation anesthesia" OR AB "Inhalation anesthesia" OR TI "Inhalation anaesthetics" OR AB "Inhalation anaesthetics" OR TI "Volatile Anesthesia" OR AB "Volatile Anesthesia" OR TI Desflurane OR AB Desflurane OR TI Sevoflurane OR AB Sevoflurane OR TI Isoflurane OR AB Isoflurane OR TI "Volatile Anaesthetics" OR AB "Volatile Anaesthetics" OR TI "Inhalational Anesthesia" OR AB "Inhalational Anesthesia" OR TI "Inhaled Anesthesia" OR AB "Inhaled Anesthesia") ) AND ( (MH "Obesity" OR MH "Morbid Obesity" OR TI Obesity OR AB Obesity OR TI "Morbid Obesities" OR AB "Morbid Obesities" OR TI Obese OR AB Obese OR TI "Severely Obese" OR AB "Severely Obese" OR TI Overweight OR AB Overweight) ) | 72 results |

**Supplementary Table 6. Cochrane Search Results**

| **ID** | **Search** | **Results** |
| --- | --- | --- |
| #1 | MeSH descriptor: [Anesthesia, Intravenous] explode all trees | 2231 |
| #2 | ((Intravenous anesthesia OR TIVA OR "Total Intravenous Anesthesia" OR "Total Intravenous Anes*" OR fentanyl OR sufentanil OR remifentanil OR "IV anesthesia" OR propofol OR "Anaesthetics Intravenous")):ti,ab,kw | 450695 |
| #3 | MeSH descriptor: [Anesthesia, Inhalation] explode all trees | 2175 |
| #4 | ((Inhalation anesthesia OR "Inhalation anaesthetics" OR "Volatile Anesthesia" OR desflurane OR sevoflurane OR isoflurane OR "Volatile Anaesthetics" OR "Inhalational Anesthesia" OR "Inhaled Anesthesia")):ti,ab,kw | 14528 |
| #5 | MeSH descriptor: [Obesity] explode all trees | 22114 |
| #6 | ((Obesity OR "Morbid Obesities" OR Obese OR "Severely Obese" OR Overweight)):ti,ab,kw | 63863 |
| #7 | #1 OR #2 | 50695 |
| #8 | #3 OR #4 | 14697 |
| #9 | #5 OR #6 | 63970 |
| #10 | #7 AND #8 AND #9 | 206 |

**Supplementary Table 7. EMBASE Search Results**

| **Search** | **Results** |
| --- | --- |
| ('intravenous anesthesia'/exp OR 'intravenous anesthesia' OR tiva OR 'total intravenous anesthesia' OR 'total intravenous anes*' OR fentanyl OR sufentanil OR remifentanil OR 'iv anesthesia' OR propofol OR 'anaesthetics intravenous') AND ('inhalation anesthesia'/exp OR 'inhalation anesthesia' OR 'inhalation anaesthetics' OR 'volatile anesthesia' OR 'volatile anaesthetics' OR isoflurane OR sevoflurane OR desflurane OR 'inhalational anesthesia' OR 'inhaled anesthesia') AND ('obesity'/exp OR overweight) | 701 results |

**Supplementary Table 8. Google Scholar Search Results**

| **Search** | **Results** |
| --- | --- |
| (TIVA OR propofol OR Intravenous anesthesia) AND (Inhaled anesthesia OR Desflurane OR Selvoflurane) AND (Obesity OR Overweight OR Obese) | 100 results |

**Supplementary Table 9. Vomiting and Nausea Subgroup Analysis**

| **Variable** | **Subgroup** | **k** | **RR (95% CI)** | **I² (%)** | **p-value (Between Groups)** |
| --- | --- | --- | --- | --- | --- |
| Inhalation | Desflurane | 4 | 2.10 [1.33; 3.30] | 42.1 |  |
|  | Sevoflurane/Desflurane | 1 | 1.86 [1.02; 3.41] | -- |  |
|  | Sevoflurane | 1 | 1.00 [0.76; 1.32] | -- |  |
|  |  |  |  |  | 0.0110 |
| Risk of Bias | Some | 3 | 1.96 [1.41; 2.74] | 0.0 |  |
|  | Low | 3 | 1.63 [0.75; 3.55] | 83.2 |  |
|  |  |  |  |  | 0.6690 |
| ASA Class | No reported | 3 | 2.01 [1.42; 2.85] | 0.0 |  |
|  | III | 1 | 3.63 [1.84; 7.14] | -- |  |
|  | I or II | 1 | 1.38 [0.68; 2.78] | -- |  |
|  | II or III | 1 | 1.00 [0.76; 1.32] | -- |  |
|  |  |  |  |  | 0.0006 |
| Surgery Type | Bariatric | 6 | 1.71 [1.17; 2.51] | 73.3 |  |
|  |  |  |  |  | -- |

**Supplementary Table 10. Vomiting and Nausea Sensitivity Analysis**

| **Author** | **Effect (95% CI: Lower; Upper)** | **I²** | **DFFITS** | **Cook's D** | **QE (del)** | **Is Influential** |
| --- | --- | --- | --- | --- | --- | --- |
| Omitting Demirel et al., 2020 | 1.64 (1.28; 2.10) | 0.706 | 1.0191 | 1.0385 | 13.59 | Yes |
| Omitting Elbakry et al., 2018 | 1.57 (1.24; 1.98) | 0.634 | 0.8575 | 0.7353 | 10.91 | Yes |
| Omitting Ziemann et al., 2014 | 1.78 (1.41; 2.25) | 0.777 | 0.3082 | 0.0950 | 17.93 | No |
| Omitting Juvin et al., 2000 | 1.83 (1.46; 2.30) | 0.786 | -0.0471 | 0.0022 | 18.67 | No |
| Omitting Honca et al., 2017 | 1.85 (1.47; 2.34) | 0.786 | -0.0382 | 0.0015 | 18.70 | No |
| Omitting Shu et al., 2024 | 2.17 (1.64; 2.87) | 0.256 | -3.7069 | 13.7411 | 5.37 | Yes |

**Supplementary Table 11. Time to Emerge Subgroup Analysis**

| **Variable** | **Subgroup** | **k** | **MD (95% CI)** | **I² (%)** | **p-value (Between Groups)** |
| --- | --- | --- | --- | --- | --- |
| Inhalation | Desflurane | 6 | 0.15 [-1.97; 2.28] | 84.6 |  |
|  | Sevoflurane | 2 | 4.96 [-4.86; 14.78] | 98.3 |  |
|  | Sevoflurane/Desflurane | 1 | 10.00 [6.54; 13.46] | -- |  |
|  |  |  |  |  | < 0.0001 |
| Risk of Bias | Some | 3 | 4.12 [-3.14; 11.38] | 97.9 |  |
|  | Low | 5 | 0.14 [-0.09; 0.36] | 0.0 |  |
|  | High | 1 | 10.00 [6.54; 13.46] | -- |  |
|  |  |  |  |  | < 0.0001 |
| ASA Class | No reported | 3 | 4.04 [-3.62; 11.70] | 93.3 |  |
|  | III | 1 | 0.80 [-1.10; 2.70] | -- |  |
|  | I or II | 2 | -1.29 [-3.82; 1.25] | 87.1 |  |
|  | II or III | 1 | 0.44 [-0.42; 1.30] | -- |  |
|  | I, II, or III | 2 | 4.90 [-4.77; 14.58] | 96.8 |  |
|  |  |  |  |  | 0.4671 |
| Surgery Type | Bariatric | 7 | 1.46 [-1.95; 4.86] | 94.3 |  |
|  | Knee arthroplasty | 1 | 0.44 [-0.42; 1.30] | -- |  |
|  | Multiple (bariatric, orthopedic, etc.) | 1 | 10.00 [6.54; 13.46] | -- |  |
|  |  |  |  |  | < 0.0001 |

**Supplementary Table 12. Time to Emerge Sensitivity Analysis**

| **Author** | **Effect (95% CI: Lower; Upper)** | **I²** | **DFFITS** | **Cook's D** | **QE (del)** | **Is Influential** |
| --- | --- | --- | --- | --- | --- | --- |
| Omitting Demirel et al., 2020 | 0.20 (-0.02; 0.41) | 0.943 | 0.2827 | 0.0799 | 123.86 | No |
| Omitting Elbakry et al., 2018 | 0.22 (-0.00; 0.44) | 0.948 | 0.0688 | 0.0047 | 135.84 | No |
| Omitting Salighoglu et al., 2001 | 0.23 (0.01; 0.46) | 0.949 | -0.0637 | 0.0041 | 136.06 | No |
| Omitting Tanaka P. et al., 2017 | 0.21 (-0.01; 0.44) | 0.949 | 0.1311 | 0.0172 | 135.94 | No |
| Omitting Juvin et al., 2000 | 0.24 (0.02; 0.45) | 0.948 | -0.0813 | 0.0066 | 133.41 | No |
| Omitting Honca et al., 2017 | 0.31 (0.08; 0.53) | 0.941 | -0.7047 | 0.4966 | 118.38 | Yes |
| Omitting Siampalioti et al., 2015 | 0.14 (-0.08; 0.35) | 0.890 | 0.8250 | 0.6806 | 63.73 | Yes |
| Omitting Mutholib et al., 2024 | 0.19 (-0.03; 0.41) | 0.934 | 0.3501 | 0.1225 | 105.51 | No |
| Omitting Aftab et al., 2019 | 0.80 (0.25; 1.35) | 0.947 | -5.1541 | 26.5648 | 131.24 | Yes |

**Supplementary Table 13. ICU Stay Duration Subgroup Analysis**

| **Variable** | **Subgroup** | **k** | **MD (95% CI)** | **I² (%)** | **p-value (Between Groups)** |
| --- | --- | --- | --- | --- | --- |
| Inhalation | Desflurane | 3 | 2.0159 [-9.2997; 13.3314] | 86.5 |  |
|  | Sevoflurane/desflurane | 1 | 0.0000 [-7.5745; 7.5745] | -- |  |
|  | Sevoflurane | 1 | 0.2600 [-0.6798; 1.1998] | -- |  |
| **p-value** | **(Between Groups)** |  |  |  | **0.9528** |
| Risk of Bias | Low | 4 | 1.7928 [-4.6908; 8.2764] | 85.5 |  |
|  | Some | 1 | 0.0000 [-7.5745; 7.5745] | -- |  |
| **p-value** | **(Between Groups)** |  |  |  | **0.7245** |
| ASA | III | 1 | 8.8200 [4.8938; 12.7462] | -- |  |
|  | II or III | 2 | -0.0875 [-1.8822; 1.7071] | 19.1 |  |
|  | No reported | 2 | -9.3709 [-45.7820; 27.0402] | 39.2 |  |
| **p-value** | **(Between Groups)** |  |  |  | **0.0002** |
| Surgery | Bariatric | 4 | 2.8322 [-3.0613; 8.7256] | 84.2 |  |
|  | Knee arthroplasty | 1 | -2.5000 [-7.2725; 2.2725] | -- |  |
| **p-value** | **(Between Groups)** |  |  |  | **0.1682** |

**Supplementary Table 14. ICU Stay Duration Sensitivity Analysis**

| **Author** | **Effect (95% CI: Lower; Upper)** | **I^2** | **DFFITS** | **Cook's D** | **QE (del)** | **Is Influential** |
| --- | --- | --- | --- | --- | --- | --- |
| Omitting Elbakry et al., 2018 | 0.147 (-0.768; 1.062) | 0.000 | 0.983 | 0.966 | 2.912 | Yes |
| Omitting Tanaka P. et al., 2017 | 0.706 (-0.202; 1.613) | 0.842 | -0.246 | 0.060 | 19.019 | No |
| Omitting Ziemann et al., 2014 | 0.602 (-0.295; 1.500) | 0.855 | -0.018 | 0.000 | 20.668 | No |
| Omitting Juvin et al., 2000 | 0.601 (-0.290; 1.493) | 0.842 | -0.017 | 0.000 | 18.986 | No |
| Omitting Shu et al., 2024 | 3.584 (0.771; 6.396) | 0.811 | -6.575 | 43.229 | 15.866 | Yes |

**Supplementary Table 15. HR Subgroup Analysis**

| **Variable** | **Subgroup** | **k** | **MD (95% CI)** | **I² (%)** | **p-value (Between Groups)** |
| --- | --- | --- | --- | --- | --- |
| Inhalation | Sevoflurane | 3 | 1.43 [-1.11; 3.96] | 0.0 |  |
|  | Desflurane | 1 | 6.10 [3.86; 8.34] | -- |  |
|  |  |  |  |  | 0.0068 |
| Risk of Bias | Some | 1 | 5.74 [-1.08; 12.56] | -- |  |
|  | Low | 3 | 2.91 [-1.46; 7.27] | 77.8 |  |
|  |  |  |  |  | 0.4929 |
| ASA Class | I, II, or III | 1 | 5.74 [-1.08; 12.56] | -- |  |
|  | III | 1 | 6.10 [3.86; 8.34] | -- |  |
|  | I or II | 1 | -1.20 [-10.99; 8.59] | -- |  |
|  | II or III | 1 | 0.90 [-1.94; 3.74] | -- |  |
|  |  |  |  |  | 0.0259 |
| Surgery Type | Bariatric | 4 | 3.49 [0.01; 6.98] | 67.6 | -- |

**Supplementary Table 16. HR Sensitivity Analysis**

| **Author** | **Effect (95% CI: Lower; Upper)** | **I²** | **DFFITS** | **Cook's D** | **QE (del)** | **Is Influential** |
| --- | --- | --- | --- | --- | --- | --- |
| Omitting Babayigit et al., 2020 | 3.94 (2.21; 5.67) | 0.778 | 0.1275 | 0.0163 | 9.02 | No |
| Omitting Elbakry et al., 2018 | 1.43 (-1.11; 3.96) | 0.000 | 3.0573 | 9.3469 | 1.95 | Yes |
| Omitting Salighoglu et al., 2001 | 4.21 (2.50; 5.91) | 0.754 | -0.1856 | 0.0344 | 8.13 | No |
| Omitting Shu et al., 2024 | 5.74 (3.65; 7.82) | 0.014 | -1.9711 | 3.8851 | 2.03 | Yes |

**Supplementary Table 17. MAP Subgroup Analysis**

| **Variable** | **Subgroup** | **k** | **MD (95% CI)** | **I² (%)** | **p-value (Between Groups)** |
| --- | --- | --- | --- | --- | --- |
| Inhalation | Sevoflurane | 3 | 7.88 [-7.65; 23.40] | 94.1 |  |
|  | Desflurane | 1 | 11.50 [7.97; 15.03] | -- |  |
|  |  |  |  |  | 0.6557 |
| Risk of Bias | Some | 1 | -0.75 [-11.42; 9.92] | -- |  |
|  | Low | 3 | 11.57 [-1.31; 24.45] | 95.2 |  |
|  |  |  |  |  | 0.1490 |
| ASA Class | I, II, or III | 1 | -0.75 [-11.42; 9.92] | -- |  |
|  | III | 1 | 11.50 [7.97; 15.03] | -- |  |
|  | I or II | 1 | 23.60 [16.45; 30.75] | -- |  |
|  | II or III | 1 | 0.53 [-2.73; 3.79] | -- |  |
|  |  |  |  |  | < 0.0001 |
| Surgery Type | Bariatric | 4 | 8.86 [-2.01; 19.74] | 93.2 | -- |

**Supplementary Table 18. MAP Sensitivity Analysis**

| **Author** | **Effect (95% CI: Lower; Upper)** | **I²** | **DFFITS** | **Cook's D** | **QE (del)** | **Is Influential** |
| --- | --- | --- | --- | --- | --- | --- |
| Omitting Babayigit et al., 2020 | 7.40 (5.12; 9.67) | 0.952 | -0.3116 | 0.0971 | 41.91 | No |
| Omitting Elbakry et al., 2018 | 4.13 (1.27; 6.98) | 0.941 | 2.5736 | 6.6235 | 33.94 | Yes |
| Omitting Salighoglu et al., 2001 | 5.27 (2.94; 7.61) | 0.906 | 1.5603 | 2.4345 | 21.27 | Yes |
| Omitting Shu et al., 2024 | 12.69 (9.65; 15.73) | 0.871 | -4.9777 | 24.7774 | 15.46 | Yes |

**Supplementary Table 19. Pain Subgroup Analysis**

| **Variable** | **Subgroup** | **k** | **SMD (95% CI)** | **I² (%)** | **p-value (Between Groups)** |
| --- | --- | --- | --- | --- | --- |
| **Inhalation Type** | Desflurane | 4 | 1.36 [-1.47; 4.19] | 98.0 |  |
|  | Sevoflurane | 1 | 0.29 [-0.22; 0.79] | -- |  |
|  |  |  |  |  | 0.4631 |
| **Risk of Bias** | Low | 5 | 1.14 [-1.08; 3.36] | 97.4 |  |
|  |  |  |  |  | -- |
| **Surgery Type** | Bariatric | 4 | 1.44 [-1.35; 4.22] | 98.0 |  |
|  | Knee arthroplasty | 1 | -0.02 [-0.43; 0.40] | -- |  |
|  |  |  |  |  | 0.3116 |
| **ASA Class** | III | 1 | 5.74 [4.84; 6.64] | -- |  |
|  | II or III | 2 | 0.10 [-0.22; 0.42] | 0.0 |  |
|  | No reported | 1 | -0.11 [-0.83; 0.61] | -- |  |
|  | I, II or III | 1 | -0.10 [-0.39; 0.19] | -- |  |
|  |  |  |  |  | <0.0001 |

**Supplementary Table 20. Pain Sensitivity Analysis**

| **Author** | **Effect (95% CI: Lower; Upper)** | **I²** | **DFFITS** | **Cook's D** | **QE (del)** | **Is Influential** |
| --- | --- | --- | --- | --- | --- | --- |
| Omitting Elbakry et al., 2018 | -0.0158 (-0.2219; 0.1903) | 0.0000 | 2.8280 | 7.9977 | 1.7186 | Yes |
| Omitting Tanaka P. et al., 2017 | 0.3608 ( 0.1310; 0.5906) | 0.9797 | -0.8763 | 0.7679 | 150.0952 | Yes |
| Omitting Juvin et al., 2000 | 0.3041 ( 0.0949; 0.5132) | 0.9799 | -0.3195 | 0.1021 | 151.3720 | No |
| Omitting Aftab et al., 2019 | 0.6115 ( 0.3332; 0.8899) | 0.9783 | -3.3410 | 11.1625 | 140.4666 | Yes |
| Omitting Shu et al., 2024 | 0.2700 ( 0.0514; 0.4885) | 0.9800 | 0.0200 | 0.0004 | 152.5762 | No |

**Supplementary Table 21. Morphine Subgroup Analysis**

| **Variable** | **Subgroup** | **k** | **MD (95% CI)** | **I² (%)** | **p-value (Between Groups)** |
| --- | --- | --- | --- | --- | --- |
| Inhalation | Desflurane | 3 | 1.69 (-1.64, 5.03) | 96.0 |  |
|  | Sevoflurane/desflurane | 2 | 4.95 (-4.64, 14.54) | 96.7 |  |
|  | **Overall p-value** |  |  |  | 0.53 |
| RoB | Low | 3 | 1.69 (-1.64, 5.03) | 96.0 |  |
|  | Some | 1 | 0.21 (-0.28, 0.70) | -- |  |
|  | High | 1 | 10.00 (6.54, 13.46) | -- |  |
|  | **Overall p-value** |  |  |  | <0.01 |
| ASA | III | 1 | 4.99 (3.89, 6.09) | -- |  |
|  | II or III | 1 | 0.29 (-1.69, 2.27) | -- |  |
|  | No reported | 1 | 0.21 (-0.28, 0.70) | -- |  |
|  | I, II or III | 2 | 4.71 (-5.38, 14.80) | 96.8 |  |
|  | **Overall p-value** |  |  |  | <0.01 |
| Surgery | Bariatric | 3 | 1.62 (-1.66, 4.90) | 97.0 |  |
|  | Knee arthroplasty | 1 | 0.29 (-1.69, 2.27) | -- |  |
|  | Multiple (bariatric 37.5%, ortho 62.5%) | 1 | 10.00 (6.54, 13.46) | -- |  |
|  | **Overall p-value** |  |  |  | <0.01 |

**Supplementary Table 22. Morphine Sensitivity Analysis**

| **Author** | **Effect (95% CI: Lower; Upper)** | **I²** | **DFFITS** | **Cook's D** | **QE (del)** | **Is Influential** |
| --- | --- | --- | --- | --- | --- | --- |
| Omitting Elbakry et al., 2018 | 0.27 (−0.15; 0.70) | 0.90 | 3.06 | 9.34 | 31.53 | Yes |
| Omitting Tanaka P. et al., 2017 | 0.92 (0.51; 1.33) | 0.97 | −0.13 | 0.02 | 92.77 | No |
| Omitting Ziemann et al., 2014 | 2.26 (1.57; 2.95) | 0.96 | −6.71 | 45.05 | 70.49 | Yes |
| Omitting Mutholib et al., 2024 | 0.77 (0.37; 1.17) | 0.95 | 0.60 | 0.36 | 66.26 | No |
| Omitting Aftab et al., 2019 | 1.11 (0.67; 1.54) | 0.97 | −1.03 | 1.07 | 87.06 | Yes |

Supplementary Table 23. GRADE Assessment

| **Certainty assessment** | | | | | | | **№ of patients** | | **Effect** | | | **Certainty** | | **Importance** | |
| --- | --- | --- | --- | --- | --- | --- | --- | --- | --- | --- | --- | --- | --- | --- | --- |
| **№ of studies** | **Study design** | **Risk of bias** | **Inconsistency** | **Indirectness** | **Imprecision** | **Other considerations** | **Inhalational** | **TIVA** | **Relative (95% CI)** | **Absolute (95% CI)** |  | |  | |  |
| **Postoperative nausea and vomiting (assessed with: Number of events)** | | | | | | | | | | | | | | | |
| 6 | randomised trials | serious^a^ | serious^b^ | not serious | serious^c^ | publication bias strongly suspected^d^ | 133/252 (52.8%) | 72/242 (29.8%) | **RR 1.71** (1.17 to 2.51) | **211 more per 1,000** (from 51 more to 449 more) | ⨁◯◯◯ Very low^a,b,c,d^ | | IMPORTANT | |  |
| **Time to Emerge (assessed with: Mean)** | | | | | | | | | | | | | | | |
| 9 | randomised trials | serious^a^ | very serious^e^ | not serious | serious^c^ | publication bias strongly suspected^d^ | 411 | 397 | - | MD **2.22 min more** (0.95 fewer to 5.39 more) | ⨁◯◯◯ Very low^a,c,d,e^ | | IMPORTANT | |  |
| **Intensive Care Unite Stay (assessed with: Mean)** | | | | | | | | | | | | | | | |
| 5 | randomised trials | serious^a^ | very serious^f^ | not serious | serious^c^ | publication bias strongly suspected^d^ | 207 | 196 | - | MD **1.55 min higher** (3.47 lower to 6.57 higher) | ⨁◯◯◯ Very low^a,c,d,f^ | | IMPORTANT | |  |
| **Heart rate (assessed with: Mean )** | | | | | | | | | | | | | | | |
| 4 | randomised trials | serious^a^ | serious^g^ | not serious | serious^c^ | publication bias strongly suspected^d^ | 127 | 128 | - | MD **3.49 min higher** (0.01 higher to 6.98 higher) | ⨁◯◯◯ Very low^a,c,d,g^ | | IMPORTANT | |  |
| **Mean Arterial Pressure (assessed with: Mean)** | | | | | | | | | | | | | | | |
| 4 | randomised trials | serious^a^ | very serious^h^ | not serious | not serious | publication bias strongly suspected^d^ | 127 | 128 | - | MD **8.86 mmHg higher** (2.01 lower to 19.74 higher) | ⨁◯◯◯ Very low^a,d,h^ | | IMPORTANT | |  |
| **Pain (assessed with: Mean )** | | | | | | | | | | | | | | | |
| 5 | randomised trials | serious^a^ | very serious^i^ | not serious | not serious | publication bias strongly suspected^d^ | 241 | 226 | - | SMD **1.14 SD higher** (1.08 lower to 3.36 higher) | ⨁◯◯◯ Very low^a,d,i^ | | IMPORTANT | |  |
| **Morphine (assessed with: Mean)** | | | | | | | | | | | | | | | |
| 5 | randomised trials | serious^a^ | very serious^i^ | not serious | not serious | publication bias strongly suspected^d^ | 287 | 285 | - | MD **2.13 micrograms higher** (2.47 lower to 6.73 higher) | ⨁◯◯◯ Very low^a,d,i^ | | IMPORTANT | |  |
| **pH (assessed with: Mean)** | | | | | | | | | | | | | | | |
| 2 | randomised trials | serious^a^ | very serious^j^ | not serious | not serious | none | 35 | 35 | - | MD **0.04 lower** (0.09 lower to 0.01 higher) | ⨁◯◯◯ Very low^a,j^ | | IMPORTANT | |  |

**CI:** confidence interval; **MD:** mean difference; **RR:** risk ratio; **SMD:** standardised mean difference

Explanations

a. Downgraded by 1 level: Most studies had 'some concerns' in bias assessment, including unclear blinding and incomplete reporting

b. Downgraded by 1 level: High heterogeneity (I² = 73%). Potential differences in study design or populations may explain inconsistency

c. Downgraded by 1 level due to large CIs that cross clinical thresholds, limiting precise interpretation of the effect

d. Downgraded by 1 level due to suspected publication bias based on funnel plot asymmetry

e. Downgraded by 2 levels: Substantial heterogeneity (I² = 94%) with unexplained variability in effect sizes.

f. Downgraded by 2 levels: Substantial heterogeneity (I² = 80%) with unexplained variability in effect sizes.

g. Downgraded by 1 level: High heterogeneity (I² = 67%). Potential differences in study design or populations may explain inconsistency

h. Downgraded by 2 levels: Substantial heterogeneity (I² = 93%) with unexplained variability in effect sizes.

i. Downgraded by 2 levels: Substantial heterogeneity (I² = 97%) with unexplained variability in effect sizes.

j. Downgraded by 2 levels: High heterogeneity (I² = 76%). Potential differences in study design or populations may explain inconsistency

Supplemental Figure 1. Funnel Plots


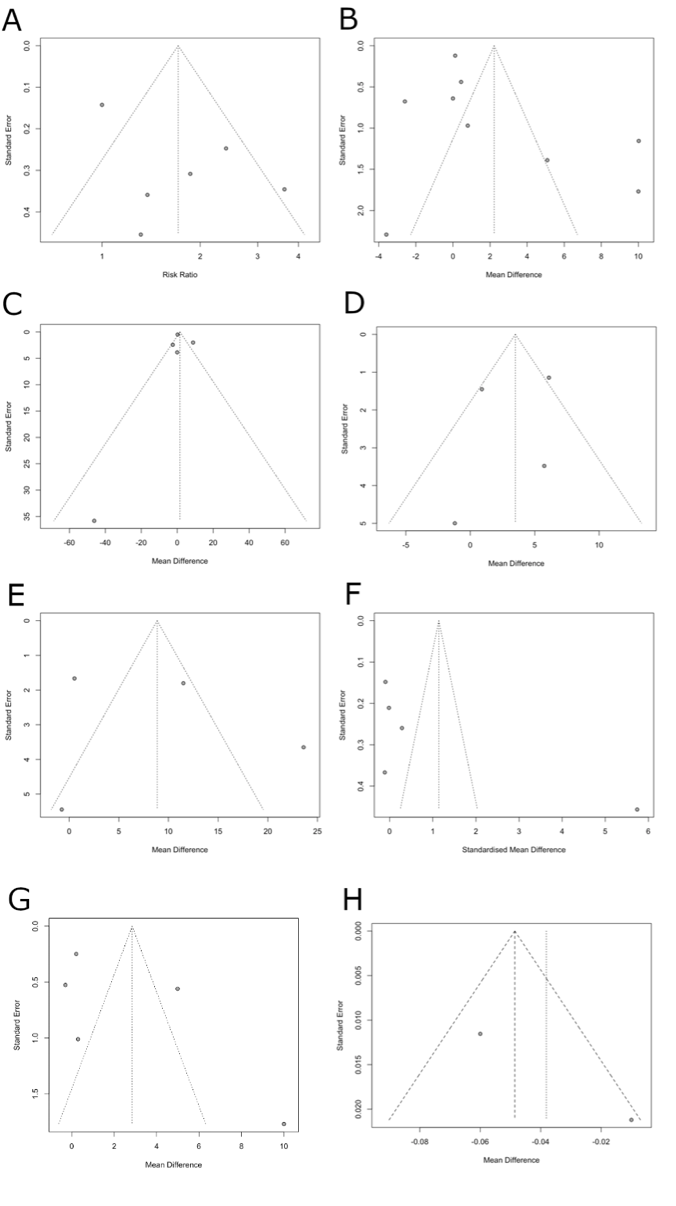


A: Vomiting and Nausea. B: Time to Emergence. C: ICU Stay Duration. D: Heart Rate. E: MAP. F: Pain. G: Morphine. H: Postoperative pH
